# Estimated preventable COVID-19-associated deaths due to non-vaccination in the United States

**DOI:** 10.1007/s10654-023-01006-3

**Published:** 2023-04-24

**Authors:** Katherine M. Jia, William P. Hanage, Marc Lipsitch, Amelia G. Johnson, Avnika B. Amin, Akilah R. Ali, Heather M. Scobie, David L. Swerdlow

**Affiliations:** 1 Center for Communicable Disease Dynamics, Department of Epidemiology, Harvard T.H. Chan School of Public Health, Boston, MA USA; 2 COVID-19 Response Team, Centers for Disease Control and Prevention, Atlanta, GA USA; 3 Department of Biostatistics and Bioinformatics, Rollins School of Public Health, Emory University, Atlanta, GA USA

**Keywords:** SARS-CoV-2, COVID-19, Vaccine-preventable deaths

## Abstract

While some studies have previously estimated lives saved by COVID-19 vaccination, we estimate how many deaths could have been averted by vaccination in the US but were not because of a failure to vaccinate. We used a simple method based on a nationally representative dataset to estimate the preventable deaths among unvaccinated individuals in the US from May 30, 2021 to September 3, 2022 adjusted for the effects of age and time. We estimated that at least 232,000 deaths could have been prevented among unvaccinated adults during the 15 months had they been vaccinated with at least a primary series. While uncertainties exist regarding the exact number of preventable deaths and more granular data are needed on other factors causing differences in death rates between the vaccinated and unvaccinated groups to inform these estimates, this method is a rapid assessment on vaccine-preventable deaths due to SARS-CoV-2 that has crucial public health implications. The same rapid method can be used for future public health emergencies.

**Supplementary Information:**

The online version contains supplementary material available at 10.1007/s10654-023-01006-3.

Vaccines for SARS-CoV-2 authorized in the United States are effective in preventing death. However, many persons remained unvaccinated after vaccines became widely available. Uptake of more recent booster doses has been limited. Several studies have estimated lives saved by vaccination [1–3]. Here, we aimed to estimate the number of laboratory-confirmed COVID-19-associated deaths that might have been averted between May 30, 2021 and September 3, 2022 if unvaccinated persons aged ≥ 18 years had been vaccinated with at least a primary series. A timely estimate of vaccine-preventable deaths could inform responses to prevent further deaths in this and future epidemics.

We used publicly available data from the U.S. Centers for Disease Control and Prevention (CDC) on COVID-19-associated deaths and death rates among individuals with a laboratory-confirmed positive test from 30 U.S. jurisdictions [4]. The 30 jurisdictions represent 68% of the total U.S. population and had a similar vaccinated proportion as the rest of the country over the period of study (Appendix I). May 30, 2021 was chosen as a start date based on when vaccines were readily available to the public, and data on deaths were only available for cases diagnosed through September 3, 2022 at the time of analysis. “Vaccinated with at least a primary series” was defined as ≥ 14 days after the series-complete dose of a COVID-19 vaccine. COVID-19-associated death was defined as a death that occurred within 30 days of a laboratory-confirmed positive test and/or was caused by COVID-19. Detailed definitions on vaccination status and COVID-19-associated deaths are provided in Appendix II. Preventable deaths in the 30 jurisdictions were estimated by multiplying the number unvaccinated by the differences in age-specific death rates among unvaccinated and vaccinated with at least a primary series, and were extrapolated to the U.S. population using the 2019 census estimate [5], after excluding the proportion of persons with an incomplete primary series. Methods are documented in Appendices II and III.

In the 30 jurisdictions, there were an estimated 158,000 (95% confidence interval [CI]: 146,000–170,000) preventable deaths among unvaccinated adults aged ≥ 18 years from May 30, 2021 to September 3, 2022 (Table 1). We extrapolated the estimate to the U.S. population after excluding persons with an incomplete primary series and estimated that approximately 232,000 (214,000–250,000) deaths in unvaccinated adults were preventable with full vaccination during the study period. Appendix IV shows the preventable deaths by age and week.

**Table 1:** Estimated number of preventable COVID-19-associated deaths among unvaccinated adults (aged ≥ 18 years), May 30, 2021–September 3, 2022

	30 jurisdictions^a^	US
Number of individuals aged ≥ 18 years (mean)	159,862,404^b^	233,656,270^c^
Estimated number of preventable COVID-19-associated deaths among unvaccinated adults (aged ≥ 18 years), May 30, 2021–September 3, 2022	158,000^d^ (146,000–170,000)	232,000^e^ (214,000–250,000)
The period of May 30–December 4, 2021	97,600 (91,000–104,000)	144,000 (134,000–154,000)
The period of December 5, 2021–April 30, 2022	52,300 (48,500–56,200)	76,300 (70,700–81,900)
The period of May 1–September 3, 2022	7,850 (6,150–9,540)	11,400 (8,910–13,800)
Age distribution of preventable deaths (%), in years		
18–29	1.38	1.37
30–49	11.9	11.8
50–64	27.3	27.7
65–79	36.5	36.1
80+	22.9	23.0

^a^The 30 jurisdictions included: Alabama, Arizona, Arkansas, California, Colorado, Connecticut, District of Columbia, Florida, Georgia, Idaho, Indiana, Kansas, Kentucky, Louisiana, Massachusetts, Michigan, Minnesota, Nebraska, New Jersey, New Mexico, New York City, North Carolina, Philadelphia, Rhode Island, South Dakota, Tennessee, Texas, Utah, Washington, and West Virginia

^b^Partially vaccinated individuals were excluded from the data set for the 30 jurisdictions; only those vaccinated with at least a primary series and unvaccinated individuals counted toward the total for each week, and this table reports the mean across the study period

^c^From the US 2019 population estimates by single year of age after excluding persons partially vaccinated

^d^COVID-19-associated death outcome was defined as a death that occurred within 30 days of a laboratory-confirmed positive test and/or was caused by COVID-19

^e^This estimate was extrapolated based on the United States 2019 population estimates for persons aged ≥ 18 years, under the assumption that the rest of the country had the same proportion of unvaccinated persons as the 30 participating jurisdictions after excluding persons partially vaccinated (proportion partially vaccinated was also assumed to be the same between the 30 jurisdictions and the rest of the country) (Appendix I)

A previous study estimated that 319,000 deaths could have been prevented by vaccination between January 1, 2021 and April 30, 2022 [6], with a monthly average of approximately 19,900. Our method implied a similar monthly estimate of approximately 20,000 per month for the period from May 30, 2021 to April 30, 2022 (Table 1), although our study started later than theirs and continued beyond April to conclude in September, 2022. Compared to the other study, we used age-stratified vaccination coverage and age-stratified death counts by vaccination status in the estimation. The 30 U.S. jurisdictions represent 68% of the U.S. population and have similar age structure, vaccination coverage, and COVID-19 cumulative incidence to the nation overall (Appendix I); therefore, data from the 30 jurisdictions are likely representative of the country. We calculated age-specific death rates by vaccination status and week to account for the changing attack rate and vaccination coverage over time. Date of SARS-CoV-2 positive specimen collection, rather than date of death, was reported in the main dataset [4]. Although there was a lag between date of test and date of death (which is noted in many other studies), the use of positive specimen collection date allowed death rates to be compared based on vaccination status at the time of infection and accounted for the potentially different time intervals between positive test and reporting of death across age and vaccination groups.

Prior infection, underlying conditions, finer age distribution within age groups, risk perception, and COVID-19 prevention behaviors may all vary between vaccinated and unvaccinated individuals and confer differing risks for COVID-19-associated death. However, data limitations prevented adjustment for these factors. Moreover, all deaths considered in the 30-jurisdiction dataset should meet the definition of COVID-19-associated deaths per national guidance [7], although some might have resulted from causes other than COVID-19. While we cannot be certain that all the preventable deaths in unvaccinated persons could have been averted by vaccination, subtracting death rates among vaccinated persons from rates among unvaccinated persons should approximate the exclusion of deaths from all causes that would have occurred even if persons were vaccinated.

Three other factors may have affected our estimates. First, the database only included laboratory-confirmed cases and deaths and might have missed deaths without laboratory-confirmed results. More generally, estimates were subject to inherent uncertainties due to misclassification in either direction of COVID-19-associated deaths, and a similar analysis using all-cause mortality might have shown a different result. Second, we underestimated deaths that were potentially preventable since vaccines became widely available for adults, as some occurred before and after the study period (May 30, 2021 to September 3, 2022). Lastly, we performed a static, week-by-week analysis comparing reported death rates in vaccinated and unvaccinated by age, thereby accounting for most secular trends related to emerging variants of concern, waning immunity, community mitigation measures, and some indirect effects related to geographic clustering of vaccinated people. The analysis estimated only the effects that could have been achieved by vaccinating unvaccinated persons, reducing their risk of infection and of death if infected, but not the effects of vaccinating these individuals on reducing transmission. A fully dynamic model incorporating these indirect effects would show a different shape for the epidemic curve in a more fully vaccinated population, as earlier vaccination of more individuals delayed infection and potentially changed the epidemic dynamics through shifting patterns of vaccine- and infection-acquired immunity [8]. However, relevant data are lacking to parameterize a model in a way that accurately accounts for prior infection by vaccination status, probability of vaccine breakthrough infection, waning immunity, and compliance with mitigation measures.

We applied a simple method as a rapid assessment using the available data to answer an important question—how many COVID-19-associated deaths among the unvaccinated could have been averted had they been vaccinated with at least a primary series? A timely answer to this question has crucial implications during a pandemic. Despite its simplicity, the current method was useful as a rapid assessment that allowed a timely estimate to illustrate the importance of vaccination in preventing further mortality during the COVID-19 pandemic. In the future, this method could be used during similar pandemic or epidemic situations to determine vaccine preventable deaths due to other pathogens.

## Supplementary Information

Below is the link to the electronic supplementary material.

Supplementary file1 (DOCX 2785 KB)

## Data Availability

Data on rates of COVID-19 deaths by age group and vaccination status are available at: https://data.cdc.gov/Public-Health-Surveillance/Rates-of-COVID-19-Cases-or-Deaths-by-Age-Group-and/3rge-nu2a.
