# Supplementary material for: Estimated preventable COVID-19-associated deaths due to non-vaccination in the United States
Source: Eur J Epidemiol. 2023 Apr 24;38(11):1125–8. doi: 10.1007/s10654-023-01006-3 (PMC10123459; doi:10.1007/s10654-023-01006-3)
Supplement: Supplementary file 1 — Supplementary file1 (DOCX 2785 KB) [file 10654_2023_1006_MOESM1_ESM.docx]

**Supplementary Appendix**

**Contents**

[**Appendix I. Characteristics of the 30 participating jurisdictions** 2](#_Toc132452922)

[**Appendix II. Definitions of vaccination status, COVID-19-associated deaths, and methodological details** 4](#_Toc132452923)

[**Appendix III. Population size and COVID-19-associated death rates among adults aged 18 years and older by vaccination status in 30 United States jurisdictions, May 30, 2021 – September 3, 2022** 6](#_Toc132452924)

[**Appendix IV. Estimated weekly preventable COVID-19-associated deaths among unvaccinated adults aged 18 years and older with laboratory-confirmed SARS-CoV-2 infection in 30 United States jurisdictions and in the United States, May 30, 2021 – September 3, 2022** 8](#_Toc132452925)

#

# **Appendix I. Characteristics of the 30 participating jurisdictions**

We compared the percentage of adults aged 18 years and older who were vaccinated with at least a primary series in 28 of the 30 jurisdictions to that in the non-participating jurisdictions, using a nationwide data set on vaccination coverage by geographic location from the United States Centers for Disease Control and Prevention (CDC, 2022a) (**Fig. S1**).


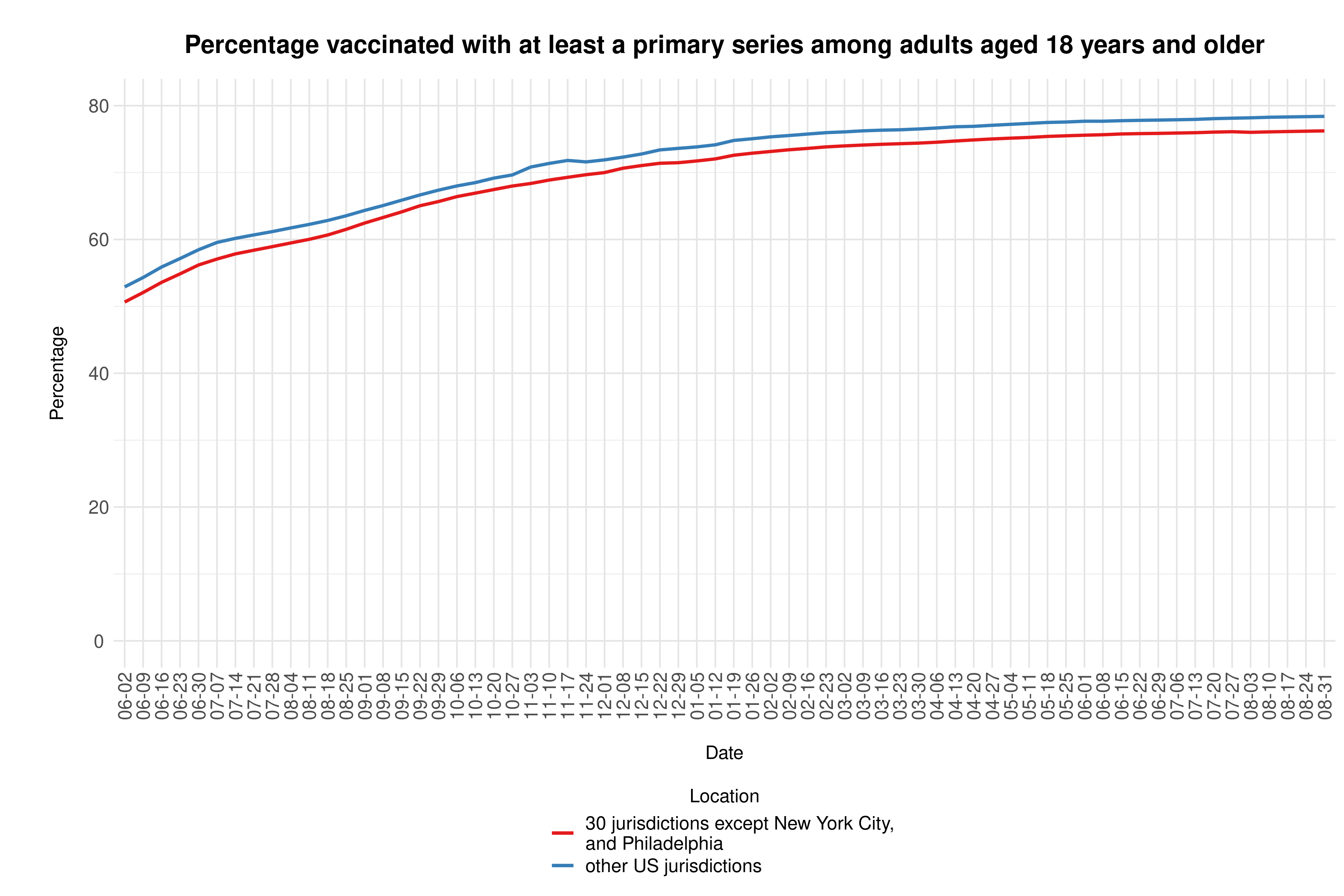


**Fig. S1** Percentage of adults aged 18 years and older who were vaccinated with at least a primary series in 28 of the 30 participating jurisdictions compared with non-participating jurisdictions, May 30, 2021–September 3, 2022. The x-axis represents the middle day of the week. Two of the 30 jurisdictions (New York City and Philadelphia) are not states and could not be identified from the data set that described vaccination coverage by state or territory (CDC, 2022a). The proportions unvaccinated and partially vaccinated are not shown. Other jurisdictions that did not report data were: Alaska, American Samoa, Delaware, Federated States of Micronesia, Guam, Hawaii, Illinois, Iowa, Maine, Marshall Islands, Maryland, Mississippi, Missouri, Montana, Nevada, New Hampshire, New York State, North Dakota, Northern Mariana Islands, Ohio, Oklahoma, Oregon, Pennsylvania, Puerto Rico, Republic of Palau, South Carolina, Vermont, Virginia, Virgin Islands, Wisconsin, and Wyoming.

In addition, the 30 U.S. jurisdictions also had similar age structure to the overall U.S. population. The overall crude incidence of COVID-19 (laboratory-confirmed) per 100 persons in the 30 jurisdictions was similar to the crude incidence in the United States (calculated based on the total reported cases per 100 persons) (**Table S1**).

**Table S1**. Comparison of 30 participating jurisdictions with the overall US population, May 30, 2021–September 3, 2022

|  | 30 jurisdictions ^a^  (CDC, 2022b) | US | Source(s) of data for US |
| --- | --- | --- | --- |
| Population |  |  |  |
| Number of individuals aged >18 years (mean) | 159,862,404 ^b^ | 233,656,270 ^c^ | (United States Census Bureau, 2021) |
| Age distribution (%), in years |  |  |  |
| 18-29 | 21.1 | 20.9 |  |
| 30-49 | 33.1 | 33.1 |  |
| 50-64 | 24.4 | 24.8 |  |
| 65-79 | 16.3 | 16.1 |  |
| 80+ | 5.01 | 5.06 |  |
| COVID-19 burden, May 30, 2021 – September 3, 2022 |  |  |  |
| Cumulative reported COVID-19 incidence | 18.8 per 100 persons ^d^ | 18.5 per 100 persons ^e^ | (Dong et al, 2020; United States Census Bureau, 2022) |
| Total number of reported COVID-19-associated deaths | 253,347 ^f^ | 457,396 ^g^ | (Dong et al, 2020) |

Abbreviations: CDC, Centers for Disease Control and Prevention

^a^ The 30 jurisdictions included: Alabama, Arizona, Arkansas, California, Colorado, Connecticut, District of Columbia, Florida, Georgia, Idaho, Indiana, Kansas, Kentucky, Louisiana, Massachusetts, Michigan, Minnesota, Nebraska, New Jersey, New Mexico, New York City, North Carolina, Philadelphia, Rhode Island, South Dakota, Tennessee, Texas, Utah, Washington, and West Virginia.

^b^ Partially vaccinated individuals were excluded from the data set for the 30 jurisdictions; only those vaccinated with at least a primary series and unvaccinated individuals counted towards the total.

^c^ From the US 2019 population estimates by single year of age after excluding persons partially vaccinated.

^d^ Cumulative incidence was calculated as the total number of new laboratory-confirmed COVID-19 cases with a positive specimen collection date from May 30, 2021 to September 3, 2022 divided by the mean population among those vaccinated with at least a primary series and unvaccinated during the period (aged 12 years old or above) in 31 jurisdictions (the 30 jurisdictions plus New York state). Please note that here the 12-17-year cases were included in the cumulative incidence only to detect any noticeably large difference compared to the national all-age incidence estimate. In all analyses, only CDC data from adults aged >18 years old and census population estimates by single year of age were used.

^e^ This estimate was obtained by dividing the total number of new reported COVID-19 cases during the study period by the US population estimates in August 2021 (including those <12 years) (United States Census Bureau, 2022). This is a crude incidence among all ages; total reported cases by age group were not available for the whole US (JHU CSSE COVID-19 Data from https://github.com/CSSEGISandData/COVID-19). All analyses for the 30 jurisdictions were conducted by age group and restricted to those aged **≥**18 years old, and estimates were extrapolated to the US population aged >18 years only, using 2019 census population estimates by single years of age (United States Census Bureau, 2021).

^f^ This number included deaths occurring among those aged >18 years with a laboratory-confirmed SARS-CoV-2-positive diagnosis and a specimen collection date from May 30, 2021 to September 3, 2022. Data are curated by CDC COVID-19 Response Team and available from the CDC (2022b).

^g^ This number included all-age COVID-19-associated deaths in the United States with a date of reporting from May 30, 2021 to September 3, 2022. Note that this is not directly comparable to the COVID-19-associated death figures (Fig. S3-4) from 30 jurisdictions (due to different age ranges, inclusion of probable COVID-19-associated death data, and use of report date rather than test date). Details on the definitions of vaccination status and COVID-19-associated deaths for the 30 jurisdictions are in **Appendix II**.

# **Appendix II. Definitions of vaccination status, COVID-19-associated deaths, and methodological details**

**Data on COVID-19-associated deaths by vaccination status**. Data on vaccination status and COVID-19-associated deaths reported to the CDC were aggregated across 30 participating United States jurisdictions (CDC, 2022b). In this study, jurisdictions were included if they regularly report linked case surveillance and immunization data to the CDC. Thirty of the jurisdictions reported deaths among vaccinated and unvaccinated persons, and were included in the analysis.

**Vaccination status**. Individuals were considered as “vaccinated with at least a primary series” 14 days following the series-complete dose of a COVID-19 vaccine authorized or approved by the United States Food and Drug Administration (FDA) (Scobie et al 2021). The number of unvaccinated persons was estimated by subtracting the numbers of persons vaccinated with at least a primary series and partially vaccinated persons (which were determined by vaccine administration data) from the United States 2019 population estimate (Johnson et al 2022).

Partially vaccinated individuals, who had at least one dose of vaccine, but did not complete the series at least 14 days before the time of record, were excluded from the data set (CDC, 2022b). Correspondingly, we excluded partially vaccinated persons when extrapolating the number of preventable deaths from the 30 jurisdictions to the United States. The weekly number of partially vaccinated individuals by age group in the 30 jurisdictions was obtained from the U.S. CDC. The overall proportion of partially vaccinated individuals in the United States is assumed to be the same as the 30 jurisdictions.

**Ascertainment of COVID-19-associated deaths**. When reporting the COVID-19-associated deaths, the 30 jurisdictions were required to follow national guidelines (CSTE, 2021). A COVID-19-associated death was defined as a person who had a documented laboratory-confirmed positive test result and died, and whose report was reviewed by local health authorities to make this determination. Per national guidance, this should include deaths among cases meeting the confirmed COVID-19 surveillance case definition and either (1) a case investigation that determined COVID-19 as cause or contributor to the death, (2) a death certificate indicating COVID-19 as a cause of death regardless of time elapsed since laboratory-confirmed positive test, or (3) a death occurring within 30 days of laboratory-confirmed positive test used to define the case and was due to natural causes. (Johnson et al, 2022). Methods of outcome ascertainment varied across jurisdictions: it was common to use vital records, while some jurisdictions used a combination of vital records and provider reporting and/or case investigations (Scobie et al, 2021).

It should be noted that the data set recorded cases and deaths among individuals vaccinated with at least a primary series and unvaccinated individuals based on the week of positive specimen collection (CDC, 2022b). Some partially vaccinated people might be infected before but received a positive test result after the 14th day following a series complete dose, but those individuals would be a small minority of cases.

**Mortality rate difference and preventable deaths**. For each week and for each age group in the 30 jurisdictions in the study period, we estimated the mortality rate difference by subtracting the rate of deaths among vaccinated individuals with lab-confirmed SARS-CoV-2 infection from that among unvaccinated individuals. This measure will account for both the changing attack rate and increasing proportion vaccinated during the period.

$$\text{Mortality rate }\text{difference}_{\text{ age group, week}}$$

$$=(\frac{\text{\# deaths among the }\text{unvaccinated }_{\text{age group, week}}}{\text{\# }\text{unvaccinated }_{\text{age group, week}}})-(\frac{\text{\# deaths among the }\text{vaccinated}_{\text{ age group, week}}}{\text{\# }\text{vaccinated}_{\text{ age group, week}}})$$

We then multiplied the mortality rate difference by the total number of unvaccinated individuals in the 30 jurisdictions by age group and week to estimate the number of preventable deaths. This calculation is equivalent to subtracting the number of deaths among the unvaccinated individuals by the number of deaths among the vaccinated individuals multiplied by the age-specific ratio of their population sizes.

$$\text{Preventable }\text{deaths}_{\text{age group, week}}$$

$$=\text{Mortality rate }\text{difference}_{\text{age group, week}}*\text{\# }\text{unvaccinated}_{\text{age group, week}}$$

$$=\text{\# deaths among the }\text{unvaccinated }_{\text{age group, week}}-\text{\# deaths among the }\text{vaccinated}_{\text{ age group, week}}*\frac{\text{\# }\text{unvaccinated }_{\text{age group, week}}}{\text{\# }\text{vaccinated}_{\text{ age group, week}}}$$

Confidence intervals were developed for the number of preventable deaths each week in each age group and overall, using Wilson’s score intervals for proportional differences.

Thirdly, we extrapolated the preventable deaths to the whole United States population, with the partially vaccinated excluded from the numerator (see formula below). Specifically, we multiplied the total United States resident population of each single year-age by the proportion (1 - % partially vaccinated) for each age group (United States Census Bureau, 2022), and then extrapolated the preventable mortality as follows:

$$\text{Preventable deaths in the }\text{United States}_{\text{age group, week}}$$

$$=\text{Preventable deaths in the 30 }\text{jurisdictions}_{\text{age group, week}}\text{ }$$

$$\text{*}\frac{\text{(1-\% partially }\text{vaccinated in the United States}_{\text{age group, week}}\text{) * Population size in the }\text{United States}_{\text{age group}}\text{ }}{\text{(Vaccinated with at least a primary series + unvaccinated in the 30 }\text{jurisdictions)}_{\text{age group, week}}}$$

$$=\text{Preventable deaths in the 30 }\text{jurisdictions}_{\text{age group, week}}$$

$$*\frac{\text{(Vaccinated with at least a primary series + unvaccinated in the United States)}{}_{\text{age group, week}}}{\text{(Vaccinated with at least a primary series + unvaccinated in the 30 }\text{jurisdictions)}_{\text{age group, week}}}$$

Lastly, we summed the weekly number of preventable deaths by age and week to obtain the total number of preventable deaths among the unvaccinated population from May 30, 2021 to September 3, 2022.

# **Appendix III. Population size and COVID-19-associated death rates among adults aged 18 years and older by vaccination status in 30 United States jurisdictions, May 30, 2021 – September 3, 2022**


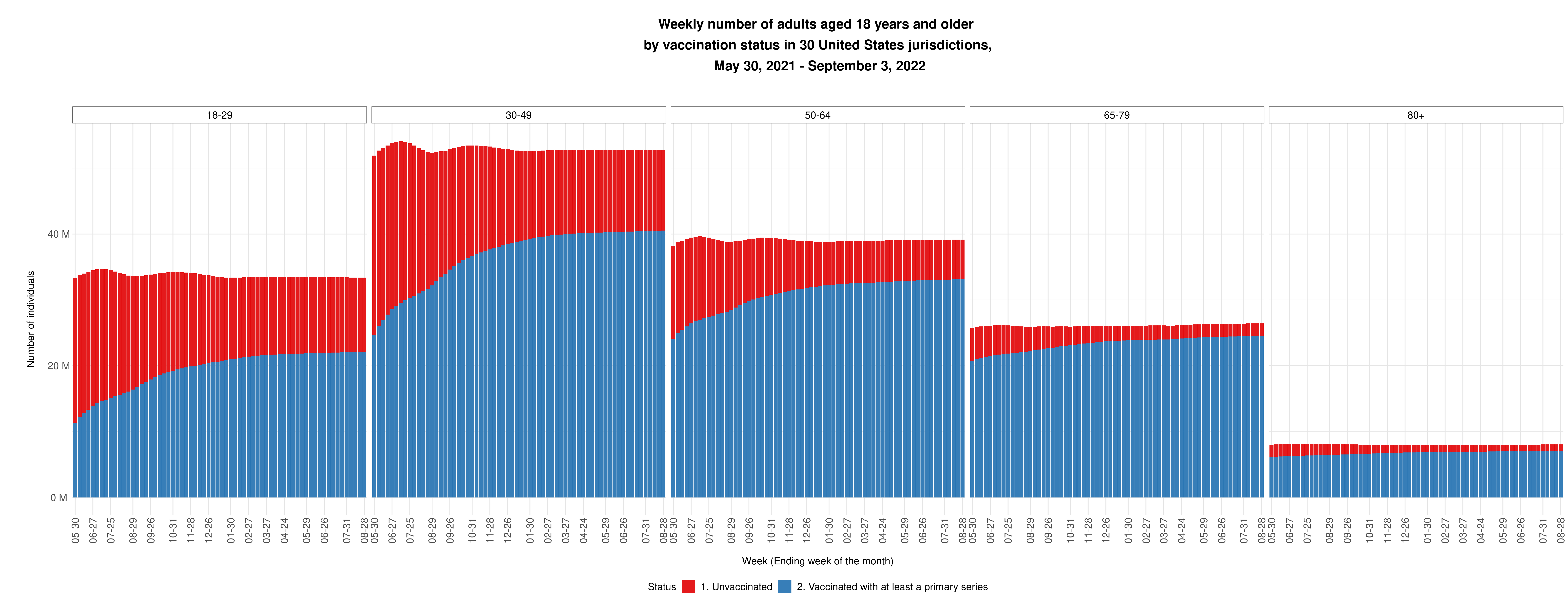


**Fig. S2** The bar plot shows the total number of individuals aged 18 and older in the 30 jurisdictions by vaccination status (partially vaccinated individuals were excluded) and age group from the Centers for Disease Control and Prevention Data Tracker (CDC, 2022b).

|  |
| --- |
| ***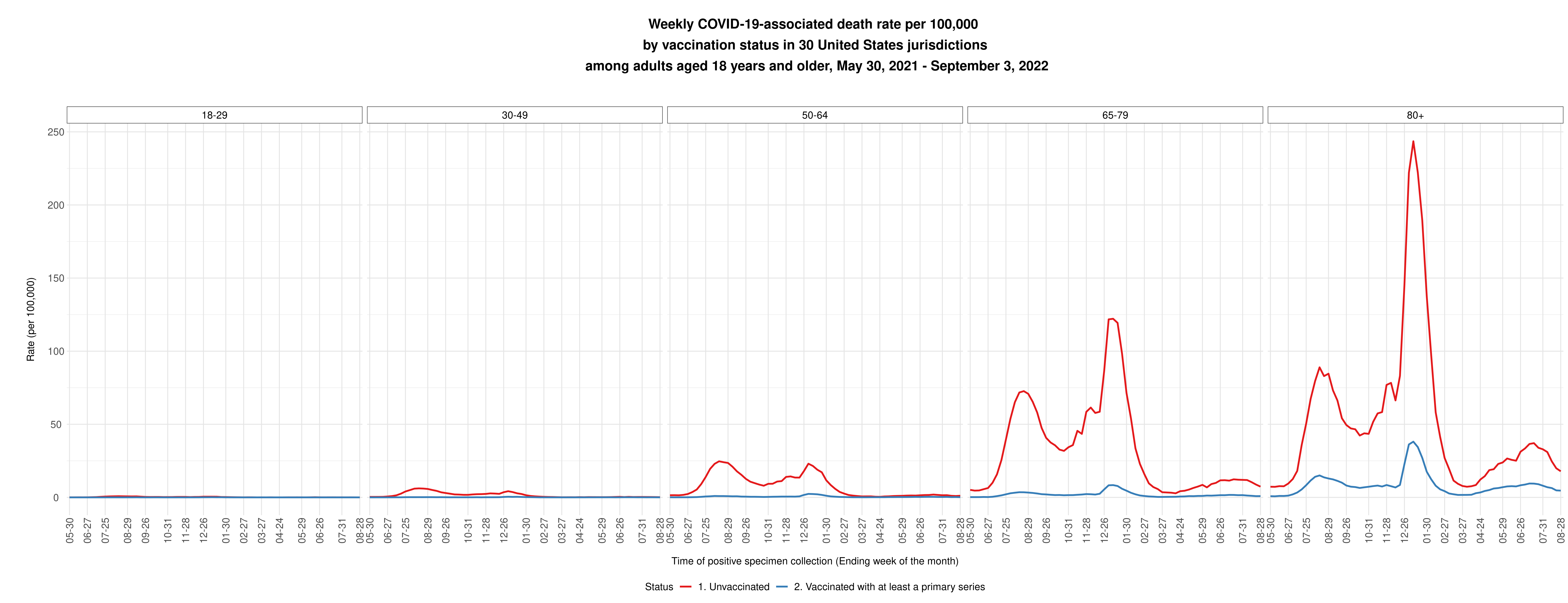*** |
| **Fig. S3** The line graph shows the mortality rate (per 100,000) for COVID-19-associated deaths in the two groups. The number of preventable deaths was estimated by multiplying the red bars in Fig. S2 by the difference between the red and blue lines in Fig. S3 (i.e., preventable death rate among the unvaccinated population) by age group and week. |

# **Appendix IV. Estimated weekly preventable COVID-19-associated deaths among unvaccinated adults aged 18 years and older with laboratory-confirmed SARS-CoV-2 infection in 30 United States jurisdictions and in the United States, May 30, 2021 – September 3, 2022**


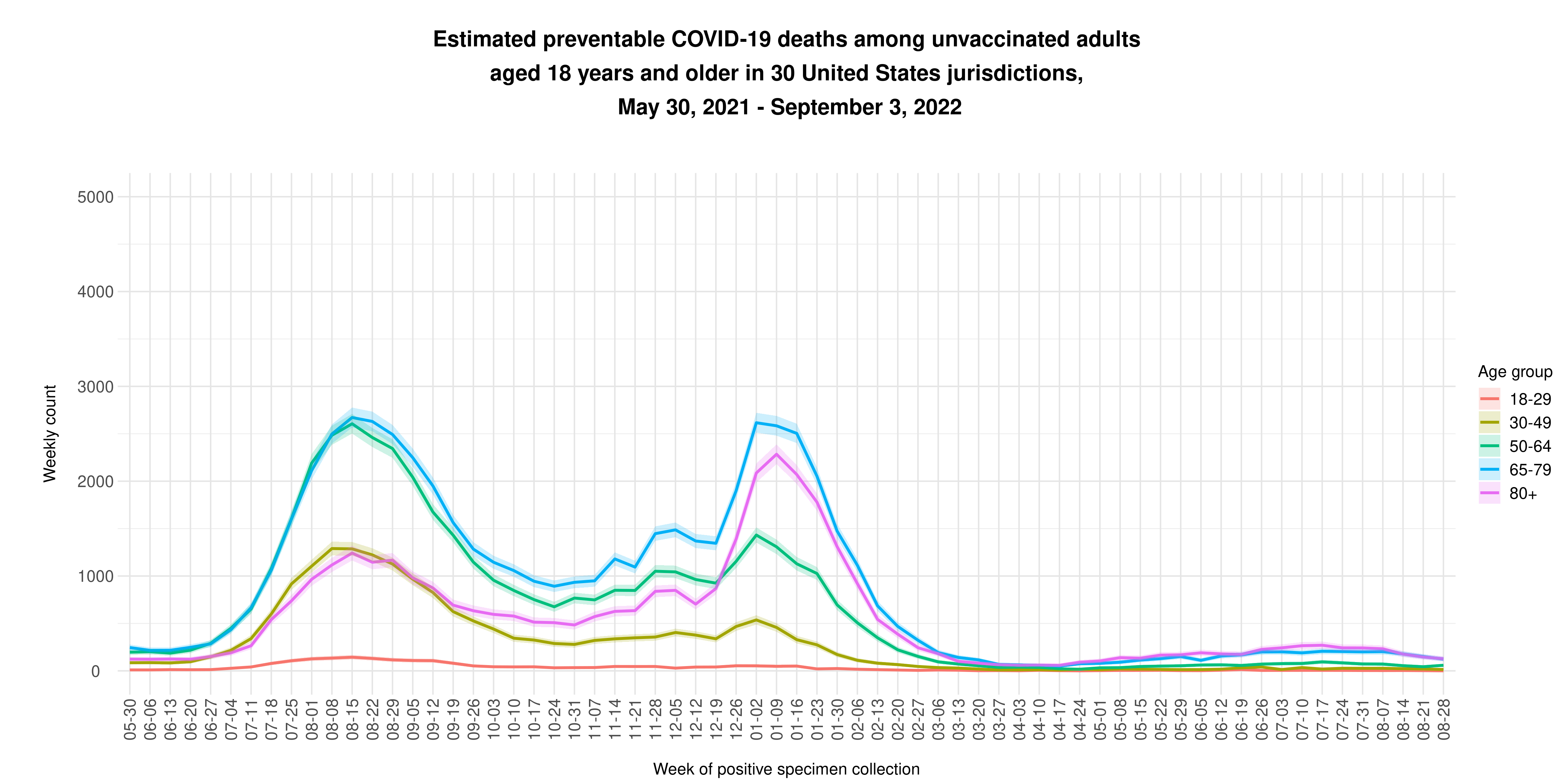


**A**

**
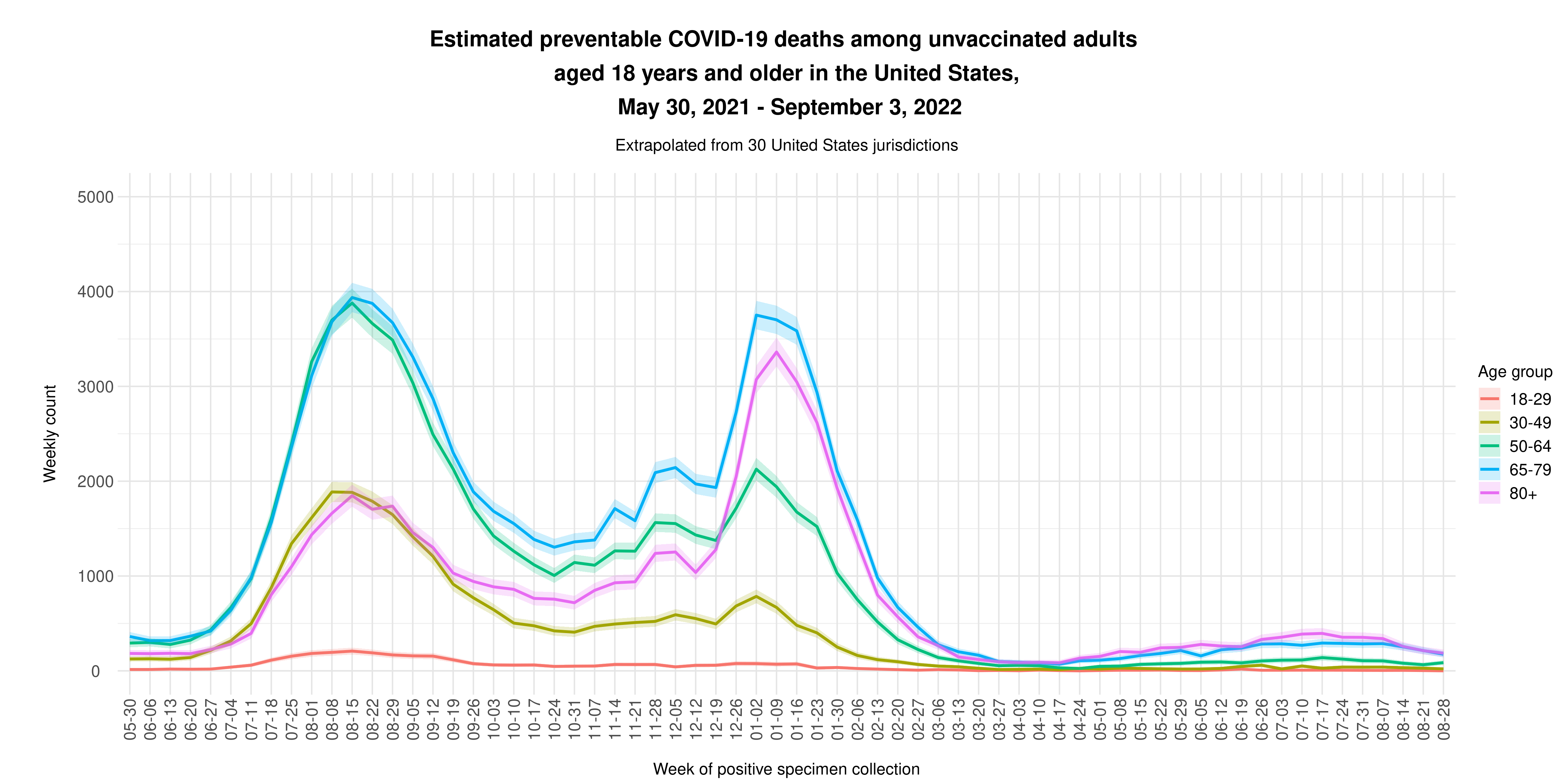
**

**B**

**Fig. S4** Estimated weekly preventable COVID-19-associated deaths among unvaccinated adults aged 18 years and older with laboratory-confirmed SARS-CoV-2 infections in (A) 30 United States jurisdictions and (B) the United States, May 30, 2021 – September 3, 2022. Shaded areas represent the 95% confidence interval.

**References for the supplementary appendix**:

Centers for Disease Control and Prevention (CDC). (2022a). *COVID-19 Vaccinations in the United States.* Retrieved 18 Apr 2023 from https://covid.cdc.gov/covid-data-tracker/#vaccinations_vacc-people-fully-percent-total.

Centers for Disease Control and Prevention (CDC). (2022b). *Rates of COVID-19 Cases or Deaths by Age Group and Vaccination Status.* Retrieved 23 March 2023 from https://data.cdc.gov/Public-Health-Surveillance/Rates-of-COVID-19-Cases-or-Deaths-by-Age-Group-and/3rge-nu2a

Council of State and Territorial Epidemiologists (CSTE). (2021). *Interim Guidance for Public Health Surveillance Programs for Classification of COVID-19- associated Deaths among COVID-19 Cases*. Retrieved 30 Mar 2022 from https://cdn.ymaws.com/www.cste.org/resource/resmgr/pdfs/pdfs2/20211222_interim-guidance.pdf

Dong E, Du H, Gardner L. An interactive web-based dashboard to track COVID-19 in real time. *Lancet Inf Dis*. 20(5):533-534. DOI: 10.1016/S1473-3099(20)30120-1

Johnson AG, Amin AB, Ali AR, Hoots B, Cadwell BL, Arora S et al. (2022). COVID-19 Incidence and Death Rates Among Unvaccinated and Fully Vaccinated Adults with and Without Booster Doses During Periods of Delta and Omicron Variant Emergence — 25 US Jurisdictions, April 4–December 25, 2021. *MMWR Morb Mortal Wkly Rep*,71, 132-138. DOI: http://dx.doi.org/10.15585/mmwr.mm7104e2

Scobie HM, Johnson AG, Suthar AB, Severson, R., Alden, N.B., Balter S et al. (2021). Monitoring Incidence of COVID-19 Cases, Hospitalizations, and Deaths, by Vaccination Status — 13 US Jurisdictions, April 4–July 17, 2021. *MMWR Morb Mortal Wkly Rep*,70,1284–1290. DOI: http://dx.doi.org/10.15585/mmwr.mm7037e1

United States Census Bureau. (2021). *National Population by Characteristics: 2010-2020.* Retrieved 28 Jan 2022 from: https://www.census.gov/programs-surveys/popest/technical-documentation/research/evaluation-estimates/2020-evaluation-estimates/2010s-national-detail.htm

United States Census Bureau. (2022). *Monthly Population Estimates for the United States: April 1, 2020 to December 1, 2022*. Retrieved 28 Jan 2022 from: https://data.census.gov/cedsci/table?tid=PEPNATMONTHLY2021.NA_EST2021_POP.
